# An Analysis of Guislain's Work on Insanity

**Published:** 1854-04-01

**Authors:** 


### AN ANALYSIS OF GUISLAIN'S WORK ON INSANITY.

(*Continued from No. XXIII., page* 436.)

### Sixth Lecture.

*Ecstasy considered as a form of mental alienation.*

This kind of mental disease is related on the one hand to melancholy, and on the other to mania, and also to acute dementia. This affection is exceedingly rare. I have but one example to show you. The term ecstasy is new as regards its application. It is not the ecstasy of novelists and poets, or that excited by certain religious ideas. That is the mystic ecstasy of Calmeil; a state so rare that I have never witnessed it. The kind of phrenoplexy I now describe has quite a different signification; it is a somewhat cataleptiform condition. It strongly approaches to melancholy or to mania. It is the intensity of the cause, the delicacy, the impressionability of the subject which most frequently give rise to the ecstatic form. The physiological phrenoplexy is observed in the man who is thunderstruck, confused, and embarrassed. In the morbid state it is a moral commotion which gives rise to ecstasy.

Is it not remarkable that the French authors have nowhere mentioned this disease? Can it be confounded with stupidity?*

In this singular affection, the functions of sensibility, of mobility, of the intelligence, all are suspended. When the disease presents itself in all its completeness, the patient looks like a statue. The muscular action is not enfeebled, but there is in the contracted muscles a certain tetanic tension. The patient has his eyes open, but does not see; if he winks, it is at very long intervals; he does not answer you, if you question him; he does not move from his place; his skin is insensible; question him during his convalescence, he will tell you he has felt nothing during his illness; he has had no ideas; he remembers nothing. Or else he will talk to you of hummings in the ears, of vertigo, or that he seemed as if he had no head.

All this, you will perceive, reveals a profound moral shock, which suspends the faculties, but which acts upon the muscular tone by stimulating and irritating it; for the muscles, as I have said, are not flaccid; they are firm, and the head is borne uprightly on the neck.

The pulse is sometimes slow, sometimes frequent. The skin is often cold and dry. The evacuations take place at long intervals.

Ecstasy is sometimes a primitive phrenopathy. Then it almost always succeeds to a cause, of which the action is sudden, as, for example, a severe fright.

In other cases it is the consequence of another kind of mental disease. It is not seldom present in the course of melancholy; and it may occur as an epiphenomenon of mania.

The ecstatic condition is distinguished from stupidity, which I shall describe in speaking of dementia. In this last there is a look of astonishment, a state of stupor; in the first there is tension of the whole system, an expression of nervosity.

Catalepsy offers great resemblances to ecstasy. But in ecstasy the disease is continuous, whereas in catalepsy it returns by fits, and leaves the intelligence intact.

The diagnosis becomes difficult when we have to deal with somnambulism accompanied with cataleptiform convulsions. But the aspect of the eyes, which are closed in somnambulists, the course of this condition which alternates with catalepsy, the sleep, the duration of this situation, which ends in a few hours to return afterwards—all this excludes the idea of the mental alienation of ecstasy.

The disease has nothing peculiar from melancholy in its course. Its invasion is usually sudden. It runs through its stages in three, seven, or nine months. More than nine-tenths of the patients recover.

### Seventh Lecture.

*On the Phenomena of Mania.*—Mania is a designation which may lead to erroneous ideas. All maniacs are not irritated, mischievous, or furious, as the word implies. There are maniacs of boisterous hilarity; there are religious, amorous, and vain maniacs. In others, the morbid exaltation is limited to the dominion of one sentiment, of one circle of ideas, of certain special faculties.

* Heinroth, Seelenstörungen, 1818. Heinroth alone has given a clear idea of phrenopathic ecstasy, by bringing it within the circle of the mental diseases recognised by him.

Thus, as a general proposition, mania is not fundamentally a state of furor: what it always is, is a mental activity, a state in which the morbid phenomena succeed each other with a certain rapidity.

I will define mania as a disease of the moral faculty, apyretic, irresistible, in which there is exaggeration, exaltation of one or several phrenic functions, characterized for the most part by a state of agitation, or sometimes by a manifestation of action or violent passions.

The most general pathognomonic character of mania consists in exaggeration, exaltation, agitation, aggressive passions. This disease generally carries with it petulance, force, power. It imparts an air of vigour, often of health, and sometimes of youth.

This condition, be it observed, is far from being at all times a complete alienation; it has its shades, its types, its degrees; it often reminds one, in the case of a naturally calm disposition, of the physiological state of another man who is naturally exalted.

*Special forms. Monomania considered in mania.*

I. *A patient affected with tranquil mania without delirium.*
*Maniacal exaltation* of M. Brierre.
*Mania—moral monomania.*
*Mania without delirium* of Pinel.

The fundamental character of this affection is a certain excitability of the moral faculty: a state of animation, of increase in the activity of the intellectual acts. It is a vesania characterized by an absence more or less complete of delirious ideas, by the absence of any notable lesion of the memory and of judgment. It is a rudimentary, initial, incomplete state; one of those singular conditions which remind us of moral insanity.

Let us make this patient talk—he will not utter a single irrational word indicating a pathological condition of the intelligence or ideas. In subjects of this kind the diagnosis must be chiefly drawn from the commemorative history; the attendants will inform us in what respect this man is insane. They will tell us that they discover his disease, not in his thoughts, but in his acts, in his desires, in his character. His family will tell us that from timid and silent, this man has become bold and talkative. The change which has come over his manner has struck and terrified his wife and children.

For the most part it is a want of activity, and also extravagant projects which characterize this vesania. Sometimes the morbid exaltation confines itself to an excess of tenderness. In some cases there is a remarkable prodigality in a man habitually parsimonious.

Sometimes mania consists entirely in a change in the care which a person takes in his toilette; sometimes in projects of marriage, frequent walks, religious habits. In some cases the entire disease consists in a more rapid elocution, in a greater emphasis, in greater boldness in the enunciation of ideas, in a disposition to defend untenable opinions, in an extreme excitability, susceptibility, and querulousness.

What indicates that this state is really a disease, is its appearance in phases, and periodically; the disorder, the agitation remarked in the pulse, the anomalous state of the appetite, the absence of sleep, or a sleep that is broken.

But the characters of tranquil mania may be so feebly traced that all the perspicacity of a man of experience is needed to appreciate them at their proper value. There are conditions in this disease in which the intellectual sphere remains absolutely intact, so that the patient preserves the consciousness of his state, and renders an account to himself of the exaltation which masters him.

Some writers have denied the reality of this condition when it is unaccompanied by any disorder of the intellectual functions; they have said: We do not understand the morbid exaltation of the desires, of the character of the man, of his passions, without admitting some aberration of judgment, some lesion of memory, of the imagination.

In a certain point of view the objections made in this respect are not devoid of truth. In the greater number of affections of this nature, the intellectual functions undergo derangements sufficiently marked without our being able to rank these disturbances in the class of delirious ideas. Tranquil hyperphrenia does not, we admit, always express a simple excitation of the domain of the sentiments, of the passions; it may be complicated with errors in the conceptions, and may have for allied symptoms a more or less marked incoherence in the ideas; it may present an overruling exaltation of one or other want: it may accompany an hysterical or convulsive state.

Many maniacs of this kind have to undergo degrading convictions before the tribunals, and have expiated in prisons misdeeds committed in the course of a moral alienation. Many homes, many families have been broken up, plunged into misfortune, through this singular disease, considered as a normal condition by the more distant relations, as a mental alienation by those who are nearer the patient. I have seen such unfortunate persons become the object of the most persevering and the most relentless revenge. I have seen husbands affected with this vesania, publicly accuse their wives of the most abandoned conduct. I have witnessed separations and divorces; and I have also seen, after some months, a year or two, passed in a state of mental exaltation, the parties recover their health and bitterly deplore their unhappy lot.

We must consider the different types:

*a.* Moral mania, appearing as a permanent phrenopathy.

*b.* A state which constitutes the prodromic or initial period of a mania of agitation.

*c.* A state which presents itself as a phase of the decline of a violent mania.

*d.* A situation which constitutes the intermediate interlucid period of several maniacal attacks, separated from each other by intervals more or less protracted.

*e.* A complete state of monomania.

On referring to statistics, I find that out of 100 admissions into this establishment, tranquil mania is met with 30 times as a permanent condition. It is the most frequent of all alienations, and in every case it presents the greatest difficulties with regard to diagnosis.

M. Lélut has said that this state is neither perfect reason nor perfect madness, but a situation in which the patient is not irrational, and does not give himself up to the excesses of a maniac. It is the mixed condition of which M. Moreau has spoken. The characters of this affection have also been described by Esquirol. They are found again in the moral insanity of Prichard. They have been described by the German phrenographers as an affective mania, as a *gemüthskrankheit.*

This initial state easily passes into complete mania.

II. A moral condition which offers a great resemblance to that just described is the *manie raisonnante* of Pinel, the *monomanie affective* of Esquirol.

I am at this moment unable to show you a patient affected with reasoning mania. In this vesania the reasoning faculties rise above the ordinary diapason of the mental faculties. The discourses of the patient are long pleadings. These maniacs display a constant tendency to engage in mental conflicts; and, what is more, these advocates of the madhouse are capable of discomfiting solid logicians. Their controversies are sometimes witty and logical in the extreme.

This morbid form is not often met with in the simple condition: it is often confounded with mania without delirium, in which the reasoning power remains intact. But in mania without delirium, although there is a certain acuteness in the expressions, a neatness in the ideas, a tendency to criticism, there is also more passion, irascibility, more disposition for contention than in reasoning mania: there are not that controversial spirit, that logic, that special exaltation of the ideas which are observed in this last.

The disease does not consist exclusively in this exaltation of the superior faculties, as Gall has said; it is seen also more or less in the disorders which characterize the acts. Besides the excitation of the intellectual faculties, the patient is a true maniac. It is for this reason that M. Brierre named this affection *madness of action* (*folie d'action*).

The study of this alienation and of mania without delirium claims all the solicitude of the physician. In the appreciation of these affections he will often have to combat against the inexperience of those whom he has to enlighten, and very often his opinion will be looked upon as a leaning which disposes him to discover madness everywhere: but commonly sad realities terminate by opening the eyes of the least intelligent, and by giving the verdict to the man of art.

III. There is a mania which I call *astute, malicious*, which offers many points of resemblance with the preceding varieties, but which, nevertheless, exhibits in its phenomena a ruling character.

It is an affection in which the patient is governed by a spirit of intrigue and malice. He is a rogue, a pilferer, a plotter. He generally evinces a disposition to organize plots and to draw the other patients into his snares. He seems to have the craftiness of the fox, and is sometimes distinguished by a great aptitude for every kind of artistic work. Most frequently he is lucid as regards his intellectual faculties.

I might bring before you several patients afflicted with this mania; but you would gain nothing by seeing or questioning them. Their answers would reveal no disorder, nothing but a certain frivolity of mind. They know so well how to calculate the bearing of their words, that they imitate the man of sound mind. Besides, I do not wish to humiliate them.

These patients stir up the weak against the strong, the subordinates against the chief. They quit the asylums, and come back; they figure before the courts; enter the prisons, and go out. In the prisons it is contended that they ought to be sent to the madhouse; in the asylum it is said that their place should be in the house of correction.

It is under the form of *tranquil monomania* that this alienation is usually manifested; but it may also assume the character of strong exaltation, and even be associated with furious mania.

I am acquainted with several girls who, at the epoch of menstruation, or just before, exhibit this kind of hyperphrenia, which assumes in some of them an acute and violent character.

I have often observed a certain periodicity in the march of this remarkable affection. Five or six months pass, during which the patients cannot be distinguished from persons of healthy mind. But in the spring, in the summer every year, or every second year, the malicious bent manifests itself, lasts a certain time, and gives way again to the normal condition.

IV. *A patient affected with the mania of theft.*

*Cleptomania.*—I often observe this state as a transitory symptom in the course of mania; I have sometimes also met with it as the radical phenomenon of this affection. It may be a compound condition or an elementary one. It may be a true monomania of theft.

The patient you see there, and who is remarkable for the freshness of his complexion, and the gentleness of his features, by his intelligent look, and his good behaviour, is afflicted with the mania of which I speak. He is employed as assistant-keeper. The disease announces itself in him by attacks of mania returning every three years, manifested each time by an irresistible inclination for theft. This patient, who is distinguished by many excellent qualities of heart and mind, among others, an ardent thirst for instruction, is a gardener; he steals the plants, money, and clothes of his companions; he baffles the vigilance of the most skilful keepers, and often succeeds in making his escape. He spends the money he has stolen, and robs the people with whom he lodges; he barters and makes exchanges, and cheats those with whom he deals. He gives himself up to every kind of larceny and depredation, and ends by presenting himself at the gates of the asylum for admission.

This fit lasts for several months, and is succeeded by long lucid intervals, during which this man conscientiously makes restitution, in proportion as his earnings will permit, for the money or other effects he may have stolen. It may be concluded that during these intervals he is entirely free from his disease.

Judge, then, of the position of the physician before the courts of justice when his advice is sought in a similar case. What are we to decide concerning this disposition for theft, permanent in some sort, existing from infancy, and following the oscillatory course of maniacal attacks?

I answer, without hesitation: the person in whom these phenomena are observed cannot be regarded as enjoying the command of his reason, although it present long lucid intervals.

This situation is not at all uncommon in pregnant women. A lady in this town was known on every occasion of pregnancy to visit the shops and commit numerous robberies. Her husband usually followed her, taking care to pay for what she had stolen.

All who have written of this species of vesania, recognise the important influence of hereditary predisposition in the development of the monomania of theft.

It is most frequently manifested under the form of tranquil mania; sometimes it is associated with a state of agitation and turbulence. It may occur in fits, and in some instances these fits are instantaneous.

V. I have observed cases of *mania* and *monomania* of *avarice.*

VI. The *mania* and *monomania* of *spending* are very frequent. From this alienation to the following phrenopathy there is but a step.

VII. *Mania ebriosa, mania crapulosa, mania à potu,* dipsomania, oinomania of Rayer (from οινος, wine).

The three following conditions lead to this state:—1. The habitual and immoderate use of fermented or alcoholic liquors. 2. The desire of drinking arising in the course of mania as a transitory symptom. 3. The excessive use of drink as the expression of a monomania in persons who are not accustomed to intoxication.

On the one hand, alcoholic liquors convey into the system a stimulating element, which acts unfavourably upon the heart and the depuratory organs; on the other hand, they influence the central system, and especially the nervous system, as agents of intellectual intoxication and perturbation.

The persons who give themselves up to these excesses are sometimes in a state of habitual mania; some are observed to become epileptic; others are directly attacked with dementia, or else this becomes developed as a consequence of mania or epilepsy. In some cases, not very infrequent, the abuse of alcoholic liquors leads to general paralysis.

Characteristic symptoms usually accompany this mental alienation. They indicate, on the one side, a state of cerebral congestion; on the other, a spinal cachexy, and a remarkable debility of the nervous system, revealed by apathy, general prostration, trembling of the limbs, alternating with a state of aggressive reaction, loquacity, complaints and accusations.

*Delirium tremens* is one of the varieties of this condition. It is a state of surexcitation which accompanies a singular tremor of the limbs. It may be classed among acute affections, but in many circumstances it belongs to the phrenopathics.

Here are two patients affected with mental alienation following the habitual use of alcoholic liquors. Everything reveals habitual sottishness. There is something quite peculiar and unhinged in their features. The skin has the tint of dirty silk, a remarkable puffiness. The dilated pupils give to the look a peculiarly lost and disagreeable expression. The pulse in one of these patients is remarkably small; they are not at all talkative. One of them is epileptic; the other is affected with tremulousness of the limbs. The condition of this latter patient has undergone amelioration since he has been here; I will even say that he has come near to the normal condition. The first has moments of great impatience, of anger, especially on the days which precede his convulsions. But these are less frequent since he has been subjected to the discipline of the house.

Drunkenness may present itself as an essential affection, that is, it may be a true morbid impulse, and constitute a monomania in all the force of the term. I first saw this affection in a music-master, who, every year, or sometimes every two years, abruptly quitted his studies to abandon himself to excessive drinking. He was at these times in a state of continual drunkenness, lasting for three or four months, until it disappeared as it were suddenly. Then this man again became averse to every excess, drank nothing but water, and avoided with extreme care everything that might compromise his health or his dignity. In one of these periods of lucidity, feeling the approach of his disease, he destroyed himself.

It is therefore important to distinguish *mania ebriosa* from the maniacal exaltation which is the sequela of habitual drunkenness. It cannot be confounded with the love of drink, which is a vice of manners. It differs entirely from these conditions; for what characterizes this affection is a morbid inclination, its appearance under the form of monomania and periodical attacks, the frequency of the pulse, the marked debility of the intelligence during the entire periods of the disease.

VIII. *Erotomania* may assume different forms, as *symptomatic erotomania, erotic monomania, nymphomania, hysteromania, erotic or uterine furor, satyriasis.*

Erotism is often nothing more than a morbid manifestation, showing itself as a symptom, more or less prominent, in the group of phenomena which characterize maniacal exaltation. This is seen in the subject before us. The look of the patient presents nothing morbid: his physiognomy has no expression of irritating passions. There is gaiety in his face, and maliciousness in his eyes. Nothing is out of order in his dress. His bearing is in every way correct. It is the speech that reveals the sentiments by which he is governed. His libidinous and disgusting discourse reveals that in him mania is complicated with sensual excitation. What we have learned concerning the first development of his disease, proves that it began by phenomena quite different from loose conversation. At the present time, when he thinks he is unobserved, he resorts to masturbation.

In many maniacal young women there may be remarked a certain genesial excitation, betrayed by equivocal words and a certain coquetry of bearing. After a certain time this sensual excitation is calmed; but in many cases it persists with the other phenomena of maniacal exaltation.

Most frequently this erotism brings on dementia; and during the course of this, whilst all the intellectual faculties disappear, erotic exaltation continues to show itself. A great number of epileptics are under the influence of a strong genesial excitation. Women affected with mania sometimes exhibit this phenomenon periodically at the epoch of menstruation.

*Erotomonomania* is an affection rarely met with in our establishments; it is not observed once in 150 admissions. This may also be a moral insanity. Morbid erotism is manifested in both sexes; it is more frequent in women than in men, in girls and widows than in married women. I have known it in pregnant women. It is more often found in persons who lead a chaste life, than in those who give themselves up to debauchery. It is seen at all ages. Sometimes erotism breaks out at the age of the suppression of the catamenia, and is evidently connected with a special state of the sexual organs. I have seen this morbid condition of the utero-ovarian organs attended by a peculiar turgescence to such an extent as to provoke an abundant secretion of colostrum in the mammary glands, such as is observed in pregnant women.

It is not uncommon to witness this morbid exaltation in women of advanced years and strong constitution. Nothing more curious than to hear the conversation of these erotomaniacs, to watch their airs, their toilet. With fingers covered with rings, dressed out in brilliant stuffs, they display the most sumptuous furniture and decorations, in the hope of attracting the men. Most frequently widows, sometimes grandmothers, these Messalinas of seventy, with their faded charms, cause the desolation and ruin of their families.

An erotomania, which I will call senile, is not rare among men. If we investigate the circumstances which give rise to this affection, we shall discover a congenital condition. A brother, sister, or uncle has been insane.

Erotomania in aged persons generally passes into dementia; but it may last for months, and even years, before undergoing this transformation.

We should form a false idea of erotomania, if we supposed that patients always behave with total disregard to decency. It is not generally so. Sometimes, especially the female patients, present nothing in their conduct to raise a suspicion of this affection. It is, therefore, under the form of a tranquil hyperphreny, and most frequently without any notable alteration in the ideas, that erotic monomania comes under our observation. In some cases, this vesania constitutes a turbulent, but rarely a furious, mania.

*Nymphomania*, the *aideiomania* of Marc (from αιδειον, genital organs), is more rare than erotic monomania. In this affection the symptoms announce a violent excitation of the sexual organs. It is from this affection that hysteromania, the furor uterinus, strictly so called, arises. Satyriasis in the male is a modification. I have not often observed either form. The following case of nymphomania is similar to one cited by Esquirol:—A young couple came to stay at an hotel. They had been married a week. It happened that at the moment of starting the lady observed the catamenia. Yielding to the entreaties of his wife, the husband, who was considerably older than she, abstained from all sexual intercourse, although they shared the same bed. Cohabitation only took place on the eighth day, and was immediately followed in the lady by complete mania, characterized by speech of extreme licentiousness and the most significant gesticulations. It was a furious nymphomania in the complete acceptation of the term.

These varieties of mania are sometimes connected with a peculiar temperament; but most frequently they are not primitive. Sometimes erotic mania succeeds to religious melancholy.

IX. *Joyous mania, monomania, chœromania* of Chambeyron, *mania saltans.* An epidemic choreomania, which has been well described, appeared in the fourteenth century (1373) in Belgium, Holland, and the provinces of the Rhine; it spread to several states in Germany. The patients haunted the churches, abandoned themselves to dancing in the most frantic manner, adorned their heads with flowers, and overran the country in bands. This affection at length assumed a convulsive form, and was called in Italy *Tarentism;* in France these patients were long called the convulsionaries of *Saint-Medard.*

X. *Amenomania, amenomonomania* is a variety of joyous mania, in which all the acts of the patient are impressed with an extreme urbanity and affability. Examples are found in every asylum.

XI. There is a *mania* characterized by *vanity*, the *mania of Narcissus.* This is usually manifested under the form of a tranquil mania, in which we behold the patient infatuated with his beauty, his charms, his wit, his dress, his talents, titles, and birth.

I ought to call to your attention, that in many varieties of mania there is observed greater or less exaltation of self-love. Maniacs in general possess a favourable opinion of everything that concerns them. They are satisfied that what they do could not be better done. They seldom find fault with their person, as is the case in melancholy: the melancholiac has an abject opinion of himself; the maniac, on the contrary, has a propensity to boast of his doings.

This disease is rarely observed under the form of *monomania.* It is often associated with paralyssiform symptoms. It also constitutes a tranquil mania, a moral insanity.

XII. *Ambitious mania, monomania.*—It is not necessary to interrogate the patient now before us, in order to learn the characters of his alienation. His attitude betrays the feelings which agitate him. He is an old captain of a troop of volunteers, who played a part in the revolution of 1830.

The true monomania of pride is a rare vesania: it is not seen here once in 300 admissions.

Ambition constitutes an element in many compound alienations. It may accompany special delirium. It is found associated with paralyssiform dementia. In this alienation the patient fancies himself the possessor of fabulous wealth; everything he sees belongs to him. This condition is quite distinct from the ambitious mania we have been speaking of; which is announced by the absence of every sign of muscular paralysis.

### Eighth Lecture.

*Religious mania, theomania, religious monomania.*—Look at the woman before us. She affects all the attitudes of a fervid devotion. Often she falls upon her knees, gets up, prostrates herself again, runs from right to left, sings religious hymns, and invokes, with a loud voice, the Virgin and the saints. If she had her way, the walls of her room would be covered with images and fancied relics. These manifestations of religious mania contrast in a striking manner with the *melancholy* of this name, as you may perceive in the two persons just brought to us. One of these expresses the sentiments of devotion with humility and fear. The other gives herself up to disordered gesticulation. There is in the first an animation in the features which is not found in this melancholic patient: this latter is extremely reserved in speech, and sober in her gests; whilst in the maniacal woman there is a certain rapture and ecstasy which fixes the attention.

These two forms, the one maniac, the other melancholic, mark a division established by M. Cérise, who admits a religious mystic form, penitent or oppressive, and a form which is expansive or contemplative.

Religious mania is far less frequent than religious melancholy.

*A case of loquacity.—Logomania, logodiarrhœa, logomonomania.* We observe this state in the patient before us. Most frequently the excitation of speech is found in the condition of symptomatic association, combined with other elements of mania or other fundamental forms, as delirium, dementia, and especially incoherence of ideas. It may, however, be manifested without disorder in the ideas, and even without sensible impairment of the conceptions. It occasionally announces a predisposition to phrenopathics; in fact, an extreme loquacity often characterizes the members of some families in which insanity is hereditary.

*Quarrelsome mania.*—I will show you a prospectus which created a great commotion among many men of rank in this country. It is a document elaborated in this establishment by a maniac, who, after an imperfect cure, published it. This is the printed prospectus:—"The Gleaner, political and literary journal. Motto: Respect for the constitution and laws of the Belgian people. The journal will appear daily: it will have the rare merit of being impartial; it will render justice to true merit; it will blast without mercy, mediocrity and bad faith in placemen of whatever rank." Nothing in this prospectus reveals disease; but everything points to a quite different man for those who knew the author before his illness. There was not in this man alienation in the true appreciation of the word; neither was there a normal condition. There existed that intermediate condition in which a man is not himself.

It is not without reason that some practitioners maintain that many persons regarded as cured of mental alienation, never are so, but that some traces of their disease always remain.

Amongst the maniacs we have yet seen, we have rarely witnessed any marked abnormal demonstrations. They are exalted; but the morbid excitation is not transmitted to the impulses. These are tranquil manias. But this condition is not invariable: it may rise to the condition of agitated mania. This is especially observed in joyous mania and in erotic mania.

*A subject affected with ambulatory mania.*—First, I will range in the number of agitated manias, *Ambulatory mania*, mania errabunda, Sylvestris. This state is not characterized by threats or fits of passion, nor by the necessity of destruction, but by an imperious want which drives these maniacs, for example, this man at my side, to be in perpetual change of place, to make excursions, and even long voyages. This form of disease may be met with as a special condition. More frequently it constitutes a symptomatic element in general mania.

*Subjects affected with agitated mania.—Insurrectional mania.* The maniac affected with this vesania waits for you with pallid lips, anger in his eyes; he apostrophizes you in the most insolent manner, with the most imperious air. He addresses the most outrageous expressions to the attendant.

I will now exhibit to you some patients who occupy the highest step in the ascending scale of gravity and violence of symptoms. These patients are affected with that *mania* which, with many other authors, I call *destructive*. It is the *mania* or *monomania furibunda, combative, homicidal, suicidal, mania, pyromania.* These morbid forms are becoming more and more rare, since the introduction of improvements into lunatic asylums. Many forms of alienation which at the present day, and under the influence of suitable treatment, remain in a tranquil condition, were formerly transformed into furious mania.

In destructive mania there is agitation, animation, irritation, anger, hatred: in other conditions there is an anxiety, a want, an idea of destruction, which is accomplished almost with indifference, with calm: it is an impulse without passion. In destructive mania there is pre-occupation, passion, violent passion.

That the most tumultuous mania may exist without perceptible disorder of of the intellectual functions, is certain. We have here numerous examples of this. Pinel has said: "We may justly admire the writings of Locke, and yet admit that the notions he has given concerning mania are very incomplete, when he regards it as inseparable from delirium. I thought like this author when I resumed my researches at Bicêtre upon this disease, and I was not a little surprised to see insane patients, who never offered any lesion of the understanding, and who were governed by a sort of instinct of fury, as if the affective faculties alone were impaired."

Groos has described *mania sine dilirio* in a work published in 1830. The works of Hoffbauer and of Marc contain some interesting facts relating to mania without delirium, in a medico-legal point of view.

There is no exaltation, no impulse, no desire, no passion, no element in the character of man which may not assume the hyperphrenic form.

Destructive mania may constitute a compound alienation, and be associated with a disorder of the ideas, with melancholy, madness, dementia. When mania is accompanied with delirious ideas, it is a *mania with delirium*. It is distinguished, as we shall see, from maniacal delirium in this—that in this vesania, the hallucinations and illusions coustitute the radical symptoms. In mania with delirious ideas, these last only occupy the second or third order in the morbid scale.

There is *a melancholic mania.*

There is *an epileptic mania.*

*A mania with madness.*

*A mania with dementia.*

---

